# Developing an interprofessional transition course to improve team-based HIV care for sub-Saharan Africa

**DOI:** 10.1186/s12909-020-02420-x

**Published:** 2020-12-09

**Authors:** E. Kiguli-Malwadde, J. Z. Budak, E. Chilemba, F. Semitala, D. Von Zinkernagel, M. Mosepele, H. Conradie, J. Khanyola, C. Haruruvizhe, S. Martin, A. Kazembe, M. De Villiers, M. J. A. Reid

**Affiliations:** 1 African Centre for Global Health and Social Transformation (ACHEST), Kampala, Uganda; 2 Division of Allergy and Infectious Diseases, University of Washington, Seattle, WA USA; 3 University of Malawi, College of Nursing, Zomba, Malawi; 4 Makerere University, Kampala, Uganda; 5 University of California, San Francisco, USA; 6 University of Botswana, Gaborone, Botswana; 7 Stellenbosch University, Stellenbosch, South Africa; 8 University of Global Health Equity , Kigali, Rwanda; 9 University of Zimbabwe, Harare, Zimbabwe; 10 Global Health Delivery, Diplomacy & Economics, Institute for Global Health Sciences | UCSF, 550 16th Street, 3rd Floor, San Francisco, CA 94158 USA

**Keywords:** HIV, Interprofessional, Quality improvement, Curriculum development, Sub Saharan Africa

## Abstract

**Background:**

With funding from the United States Health Resources Service Administration (HRSA), a consortium of health professional training institutions from Africa developed HIV-specific, interprofessional, team-based educational resources to better support trainees during the transition period between pre-service training and professional practice.

**Methods:**

Ten faculty members representing nine medical and nursing schools in sub-Saharan Africa (SSA) developed a training package of modules focused on core clinical, public health, interprofessional education (IPE), and quality improvement (QI) domains related to HIV service delivery. Curriculum development was informed by a rapid needs assessment of existing tools and future needs for HIV education across 27 SSA health professions training institutions. A total of 17 modules were developed, targeted at newly qualified health care professionals to be taught in a series of two-day workshops meant to complement existing institution specific HIV-curricula.

**Results:**

Between April and July 2019, a comprehensive case-based HIV training package was developed to support trainees in transition from pre-service training to independent professional practice. Each module, addressing different elements of interprofessional practice, was intended to be delivered in an interprofessional format. Thus far, 70 health professions training institutions in 14 countries have implemented the program; 547 educators facilitated STRIPE workshops, with a total of 5027 learners trained between September 2019 and September 2020.

**Conclusions:**

To our knowledge this is the first IPE HIV-specific curriculum explicitly focused on enhancing the quality of training provided to graduating health care professionals working in SSA. The collaborative, cross-institutional, interprofessional approach to curriculum development provides a benchmark for how best-practice approaches to education can be disseminated in SSA.

**Supplementary Information:**

The online version contains supplementary material available at 10.1186/s12909-020-02420-x.

## Background

Tremendous progress has been made to end the HIV epidemic in sub-Saharan Africa (SSA); unfortunately, HIV remains responsible for 660,000 deaths each year [1]. To maintain gains made to date, countries with a high HIV burden require a skilled health professional workforce to deliver sustainable, high quality care. The Medical Education Partnership Initiative (MEPI) and the Nursing Education Partnership Initiative (NEPI), both funded by the United States President’s Emergency Fund for AIDS Relief (PEPFAR) and the National Institutes of Health (NIH) between 2010 and 2015, exemplify how targeted investment of resources have led to improvements in pre-service training, retention of health care workers, and the provision and enhancement of research capabilities [2].

Despite advances made with programs such as MEPI and NEPI, gaps persist in the development of the health professional (HP) workforce in many SSA countries [3]. To bridge these gaps, SSA countries need to optimize the quality of health services delivered, recognize the synergy that effective team-based care affords, and support trainees in the vulnerable transition from school to practice, when habits in professional practice are first developed and mentorship is often lacking [4, 5]. Investment in high-quality pre-service training is also motivated by the goals of achieving epidemiologic control and consolidating existing investments. To this end, PEPFAR has repeatedly emphasized the importance of training the next generation of health professionals as crucial to ensuring the sustainability of HIV programs that were implemented over the prior 15 years [3]. Sustainability also takes into account the issue of socially accountable health professions education (HPE) that can only be achieved by addressing the needs of the communities the students will serve. The need to ensure relevance, quality, cost effectiveness, and equity for the populations must be put into consideration [6, 7].

Interprofessional training modalities that can enhance team-based HIV care, while also enabling efficient use of training resources, are likely to be of high impact in resource-constrained high HIV burden settings. Furthermore, targeting training resources at early career professionals, especially at the time of transition from pre-service training into independent professional practice, is also likely to be of high-impact, given the importance of this crucial transition period in the professional development of early career health professionals [8].

**Fig. 1:**
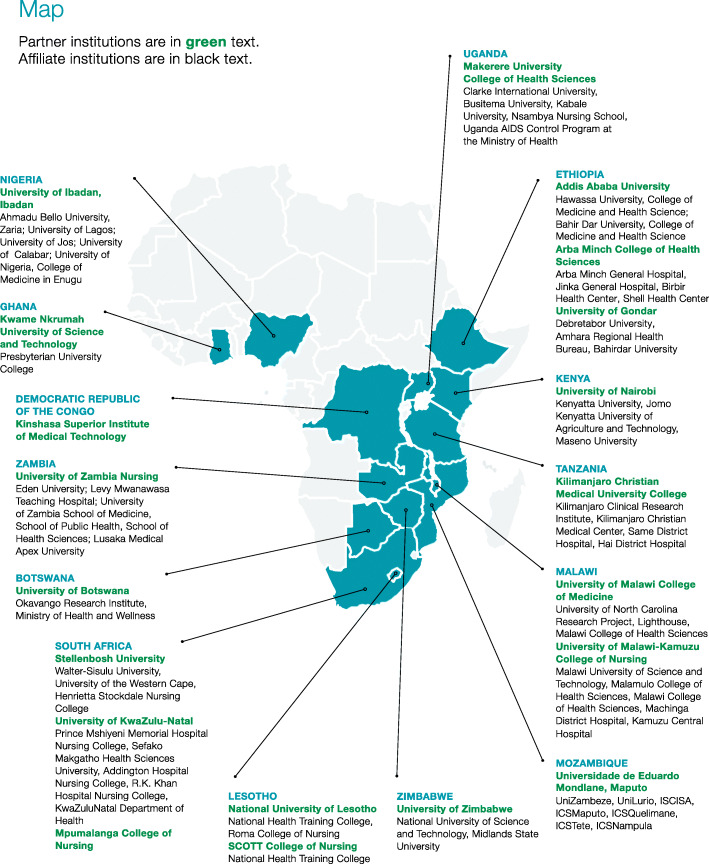
Map of partner institutions and affiliated academic training sites. Partner institutions are in green text, Affiliate institutions are in black text

To strengthen interprofessional HIV training for health care professionals, the network of African nursing and medical schools, (AFREhealth), in partnership with the University of California, San Francisco and with funding from the U.S. Health and Resources Services Administration (HRSA) implemented a novel approach to HIV training across 20 HP training institutions and their affiliated partner institutions in 14 SSA countries in 2019 (Fig. 1). Here we describe how this interprofessional approach to HIV training and health care, targeted at SSA health professionals in the transition between pre-service training and professional practice, was developed through a cross-country collaboration of health educators. In addition, we highlight how this training program was received by educators and its impact on their confidence to deliver inteprofessional education. We highlight the different components of the curriculum development process, so that our approach can serve as a useful model of evidence-informed interprofessional instructional design [9].

## Methods

### Needs assessment

Given the paucity of Africa-based literature on interprofessional curricula to improve HIV training for pre-service learners, we first surveyed health professionals from a subset of African HP training institutions affiliated with the AFREhealth network (*n* = 30). Twenty-seven institutions responded (10 medical schools, 7 nursing schools, 5 allied health professionals training institutions). Of the 27 institutions, 81% (*n* = 22) taught their students about HIV prevention, 89% (*n* = 24) clinical management including antiretroviral therapy (ART) initiation and management, and 85% (*n* = 25) pediatric HIV. Less than 65% (*n* = 17) of institutions trained graduates to integrate knowledge of interprofessional practice and/or the roles and responsibilities of different health professional cadres in clinical decision making, and only 26% (*n* = 7) introduced any kind of quality improvement training into clinical training. Furthermore, only 22% (*n* = 6) of institutions provided any kind of clinical support or resources to recent trainees after they graduated from pre-service training.

In addition to the survey, key informant interviews, conducted with 15 individuals identified as experts in health education from across SSA, and members of the AFREhealth network, highlighted key themes included lack of support for HPs after graduation and lack of recognition of large gaps between the HIV content learners were being taught prior to graduation and the breadth and quality of HIV care they were expected to deliver post-graduation.

### Expert panel and curriculum development

Informed by the survey data and key informant interviews, leading HP educators from across the AFREhealth network were invited to participate in a multi-disciplinary, multi-institution collaborative planning panel of HP educators from across the AFREhealth network. Twenty-one HP educators applied; applications were reviewed by an independent panel of health educators from the US-partner institution (UCSF), and nine were chosen to work alongside three educators from UCSF. In reviewing applicants, the independent panel prioritized selection based on ensuring an equitable mix of professional cadres and gender, as well as ranking applicants based on experience or expertise in teaching IPE or HIV content. Of the 12 who made up the expert panel, seven were physicians and five were nurses; eight were women and four were men. Five were experts in HIV. Nine medical and nursing schools from six SSA countries were represented.

The panel met in-person three times over a three-month period to develop an interprofessional curriculum, targeted at learners in the transition between pre-clinical and clinical training, to be deployed across pre-service HP training institutions in SSA. At the first convening, the team determined 17 HIV-specific topics that reflected commonly faced clinical or programmatic challenges (Table 1) and were determined to be issues of high programmatic priority for PEPFAR, based on PEPFAR’s annual reports and funding priorities. Seventeen training modules were then developed, informed by an evidence-based, step-wise process [10]. For each module, goals and objectives were determined; then, educational strategies and evaluation tools were applied to match the goals and objectives. All content was developed to optimize interprofessional engagement and intended to be delivered as 80–120 min case-based, interactive, small group workshops. The workshops are intended to be composed of learners from different cadres learning together in groups of 4–8 with oversight and instruction from facilitators from different cadres, with a suggested facilitator to learner ratio of 1 to 8–12. Facilitator guides for each module were also developed, including tools to enable effective group discussion and providing answers to all questions included. The materials (facilitator and learner guides, evaluations, and supplementary materials) are housed on a website and available for download (http://stripe.afrehealth.org). All partner schools were encouraged to adapt content to their different clinical settings and country guidelines and practice.

**Table 1:** Summary of modules included in the interprofessional curriculum

Module	Topic	Commonly encountered clinical challenges	Module goal
1	New HIV diagnosis and ART initiation in a woman of child-bearing age	Learners may not be aware that ART is recommended for everyone, regardless of CD4 cell count. In addition, learners may not recognize the urgency of starting ART given the potential for transmission to the fetus. Last, disclosing a positive result to a patient may be challenging.	The goal of this session is to prepare learners to assess and manage a woman newly diagnosed with HIV using a team-based approach.
2	Co-morbidities in a patient with well-controlled HIV	As persons with HIV live longer, learners may overlook chronic disease management, especially cardiovascular disease, given its associations with both HIV and some ART.	The goal of this session is to prepare learners to evaluate for, prevent, and manage cardio- metabolic complications in people with HIV. The session exemplifies team-based approaches to chronic disease management.
3	Management of HIV-TB co-infection	Challenges in the management of HIV-TB co-infection can arise during diagnosis, TB and ART treatment initiation, including drug-drug interactions, side effects, and timing of ART, and recognition of the stigma surrounding TB.	The goal of this module is to prepare learners to provide team-based care and follow up for a patient with HIV and pulmonary TB. This module exemplifies role clarification and use of evidence-based medicine/country-specific guidelines.
4	Prevention of mother-to-child transmission & care of the pregnant woman with HIV	Learners may not recognize the risks of and steps in prevention of HIV transmission during pregnancy and breastfeeding. In addition, selection of ART for a pregnant woman can be challenging.	The goal of this module is to prepare learners to manage the care of pregnant women, new mothers, and newborns with HIV using an integrated approach to service delivery.
5	Care of the adolescent girl at risk for HIV	Challenges include recognition of reasons why young women/adolescents may engage in sexual activity that increases risk for HIV, strategies to prevent HIV acquisition, and tactics to engage youth in their own health care.	The goal of this module is to prepare learners to recognize the unique aspects of caring for adolescent girls at risk for HIV and to provide team-based care for this patient population.
6	Post-exposure prophylaxis	Challenges in post-exposure prophylaxis (PEP) include providing support for a colleague who has had an occupational exposure to a blood borne pathogen, performing a risk assessment based on the exposure, and deciding if PEP is appropriate.	The goal of this module is to prepare learners to assess and manage any colleague who presents with a potential work-related exposure to HIV. This session exemplifies health care professionals caring for each other’s physical and mental health and highlights interprofessional competencies.
7	Care of the patient with cryptococcal meningitis	Common clinical challenges in the care of a patient with cryptococcal meningitis include obtaining a diagnosis, formulating a treatment and follow-up plan, managing increased intracranial pressures, and deciding upon when to start ART.	The goal of this session is to prepare learners to provide team-based care for a patient with cryptococcal meningitis (CCM). In addition, this module will model team members of all levels speaking up and being heard, treating patients and coworkers with compassion and respect, and using evidence-based medicine and guidelines to provide high quality care.
8	Management of sepsis and ART initiation	Common clinical challenges encountered in a patient with bacterial sepsis center on initiation of appropriate therapies in a timely manner and communicating effectively with other members of the healthcare team to do so.	The goal of this session is to introduce learners to the provision of team-based care for a patient newly diagnosed with HIV who is admitted to the hospital with bacterial sepsis.
9	ART adherence and evaluation of virologic failure	Learners may have difficulty eliciting an adherence history and also in counseling a patient on how to improve their adherence. In addition, decisions regarding ART can be difficult when it is unclear if the virologic failure is due to non-adherence or resistance mutations or both.	The goal of this session is to prepare learners to use evidenced-based strategies to provide team-based care for a patient with ART non-adherence. The session will highlight respectful communication and collaborative leadership and employ reflective practice.
10	End-of-life care in a patient with HIV	Learners may not feel comfortable breaking bad news to the patient, nor may they feel confident in how to transition medical care to ensure the comfort of a dying patient. Other common challenges include eliciting patient and family preferences for care, supporting the patient’s family, and addressing the learners’ own feelings about the end of life.	The goal of this session is to introduce learners to practice breaking bad news and to provide com- passionate, person-centered, team-based care for patients with HIV with severe life-threatening dis-eases. This session will focus on use of reflective practice and will explore the ethical dimensions in providing care to someone at the end of life, with hopes to convey the importance of compassion.
11	Pre-exposure prophylaxis and care of men who have sex with men	Common clinical challenges include taking a sexual history and eliciting potential indications for pre-exposure prophylaxis (PrEP) whilst ensuring that the healthcare professional is non-judgmental and is cognizant of stigma the patient may be experiencing.	The goal of this session is to prepare learners to provide and promote equitable HIV services to key populations using a multidisciplinary approach, doing so with empathy and without prejudice, and in the process, acquiring skills in and increasing comfort with taking a sexual history.
12	Care of the adolescent male with perinatal HIV	Learners may not know how to provide psychosocial support to an adolescent patient or a patient with perinatally-acquired HIV, including the potential need to discuss potential mental health issues and substance use. In addition, patients may have drug-resistant HIV, which can pose challenges for ART selection.	The goal of this session is to introduce learners to concepts around care of an adolescent male with perinatally-acquired HIV, so as to recognize contextual factors to best support these patients and promote their quality of life.
13	Health systems in HIV care	Common clinical challenges include managing the logistics of a drug stockout and its effect on patients’ health, including their mental health.	The goal of this module is to enable learners to better understand the building blocks of the health system and how they affect the care of the patient with HIV.
14	Community-based HIV care service delivery	Common clinical scenarios include recognition of challenges patients may face based on the health system level they are engaging with, how to manage that, and how to harness the support of non-governmental organizations and community-based initiatives.	The goal of this session is to empower learners to collaborate with community partners, understand-ing the resources, structures, and processes avail-able to patients with HIV, while also underscoring the important role of community-based, decentral-ized care to ensure patient-centered services.
15	Traditional & complementary medicine & Pneumocystis pneumonia	Common clinical challenges include difficulty in eliciting patient’s history of engagement with traditional and complementary medicines and how to build trust between the healthcare system and the patient.	The goal of this session is to prepare learners to provide person-centered care for a patient with an opportunistic pneumonia who is interested in traditional & complementary medicine (T&CM). The module will model respectful communication and reflective practice.
16	HIV funding mechanisms & donor financing	Common clinical challenges include ensuring the health care workforce stays healthy, through infection control, especially when there are already scarcities in the healthcare workforce. Other clinical challenges include attrition in the healthcare workforce.	The goal of this module is for learners to recognize human resource challenges faced in delivering high quality care, at both clinical and systems levels, and to gain an awareness of ensuring the necessary skills to deliver high quality HIV care. This is exemplified through the vignette of a health care professional with absenteeism due to nosocomial TB, which highlights certain aspects of infection control.
17	Care of the paediatric patient with HIV	Common clinical challenges in the care of a paediatric patient with HIV include diagnosis, ART management and opportunistic infection management. In addition, learners may need training in how to manage disclosure, confidentiality, and stigma.	The goal of this session is to introduce learners to care for a paediatric patient with HIV, including concepts of medication dosing, interaction with the parent/family, and ethical issues surrounding this key population.

Each panelist elaborated on two modules, and weekly Zoom© (San Jose, California) meetings were held over a two-month period to provide feedback on each module. At a second meeting, each module was reviewed again; feedback regarding clarity, flow, and applicability was incorporated and both interprofessional and quality improvement focuses ensured (Table 2). Thereafter, all modules were peer-reviewed by independent topic experts from elsewhere in Africa and the US. Peer reviewers provided rigorous assessment of both pedagogical and technical elements. The expert panel met one final time, to share the training tools with educators from 20 academic institutions from across SSA, at the annual AFREhealth symposium. (These 20 institutions were chosen by HRSA based on their participation in earlier MEPI and NEPI programs [2].) This meeting provided the opportunity to acquaint others with the curriculum and to provide explanation as to how training of facilitators and training of learners could be implemented. Facilitator training was undertaken by each individual institution, with support from the expert panel, and included ‘train-the-trainers’ (TOT) sessions and supplemental IPE webinars. To assess the impact of STRIPE HIV on educators’ confidence and aptitude to deliver IPE, educators were asked to complete an online survey consisting of quantitative and qualitative questions after facilitating workshops. This survey was developed exclusively for this anlysis; the full questionnaire is is included as a supplemental file.

**Table 2:** Interprofessional education principles and quality improvement techniques incorporated into curriculum

Interprofessional education principles^a^	Application	Quality improvement techniques	Application
Values and ethics for interprofessional practice	Construct a patient-centered management plan to ensure collaboration with the patient and all health care workers	Plan-Do-Study-Act (PDSA) Cycles^b,c^	Describe an intervention to reduce the risk of occupational exposure to HIV
Roles and responsibilities	Describe the role of all team members who provide care for the patient with cryptococcal meningitis	Fishbone (Ishikawa) Diagram^d^	Ascertain causes leading to low early infant diagnosis rates
Interprofessional communication	Use the two-challenge rule^e^ if a health professional recognizes a safety breach that is ignored by the team-leader	A3 Approach^f^	Use multiple QI modalities to address gaps in TB infection control measures
Teams & teamwork	Describe a shared decision-making approach among the patient and team members to address causes of non-adherence	5 Whys^c^	Investigate why an HIV RNA result may have been lost
		Situation-Background-Assessment-Recommendation (SBAR)^d,e^	Prepare an SBAR communication to describe a patient with cryptococcal meningitis to another healthcare professional

^a^Interprofessional Educational Collaborative. (2016). Core competencies for interprofessional collaborative practice: 2016 update. Washington, DC: Interprofessional Education Collaborative

^b^The Deming Institute. PDSA Cycle. https://deming.org/explore/pdsa/

^c^ACT Academy. Plan, Do, Study, Act (PDSA) cycles and the model for improvement. https://improvement.nhs.uk/documents/2142/plan-do-study-act.pdf

^d^Institute for Healthcare Improvement. Patient Safety Essentials Toolkit https://phpa.health.maryland.gov/mch/Documents/HV/COVID-19/IHIPatientSafetyEssentialsToolkit%20(1).pdf

^e^**TeamSTEPPS**®: Strategies & Tools to Enhance Performance and Patient Safety. [Falls Church, VA]:Dept. of Defense, TRICARE: Agency for Healthcare Research and Quality, 2010

^f^Primary Care Quality Improvement (QI): Lean Problem-Solving Tool. Content last reviewed November 2018. Agency for Healthcare Research and Quality, Rockville, MD. https://www.ahrq.gov/evidencenow/tools/qi-lean-problem-solving.html

The protocol for this project was reviewed and approved by the University of California, San Francisco’s Institutional Review Board (IRB) in San Francisco, California. Consent to participate in the facilitator survey was considered implied by participation in the study as approved by the IRB (#19–28,050).

## Results

Between April and July 2019, this comprehensive case-based HIV training package was developed to support trainees in transition from pre-service training to independent professional practice. Each module was intended to be delivered in an interprofessional format and to address different elements of interprofessional practice (namely, roles and responsibilities, values and ethics, teams and teamwork, and interprofessional communication). In addition, all modules focused on introducing learners to quality improvement tools relevant to HIV service delivery in SSA.

Between July 2019 and September 2020, 20 pre-service institutions and 50 affiliated partner institutions (Fig. 1) implemented the training program, with 547 health professions educators participating in 44 TOTs and 5027 learners attending STRIPE workshops. A subset of these educators completed all or a portion of a facilitator survey (12%, *n* = 65/547). The majority of those surveyed reported they now felt “extremely confident” in: (i) facilitating workshops as part of a team of interprofessional educators (82%, *n* = 53/65), (ii) training different types of health profession students together on HIV interprofessional collaboration (85%, *n* = 55/65), and (iii) integrating HIV IPE into their teaching practice in the future (75%, *n* = 49/65). In response to open-ended questions, the predominant theme emerging from educators was increased motivation to engage in more multidisciplinary approaches to HIV education in the future. The impact of the training, on learner knowledge and confidence, is the subject of a separate report which includes an assessment of the learners’ knowledge, confidence, and comfort with HIV and interprofessional collaboration. Subsequent analyses will be based on narratives, reflections, and focus group discussions both immediately after the workshop, but also at specific time points after the workshop.

## Discussion

To our knowledge, this is the first time an interprofessional HIV training curriculum has been adopted at this scale across African pre-service institutions. Furthermore, it is unique in its focus on learners that are in transition between pre-service and independent professional practice. This interprofessional approach to curriculum development and pre-service learning offers a model that can be used to enhance training in other areas, topics, or disciplines of pre-service training in Sub-Saharan Africa.

### Focus on transitional training

While transitional training has been increasingly adopted in diverse health disciplines, the vast majority of transition courses have focused narrowly on surgical or other procedural skills [11]. Furthermore, while curricula have been implemented to address the transition from student to resident physician in the US and Europe, this is the first time that a training resource like this has been developed for trainees from different cadres and in an African setting. In many settings in SSA, there are limited systems in place to support health professionals once they leave full time education. Moreover, the licensure requirements of different cadres in different countries are variable, such that newly graduated health care professionals in various settings practice HIV care with sub-optimal oversight, support, or continuing professional development opportunities [12]. In such contexts, transition-specific education interventions can be an effective means of enhancing clinical practice by targeting learners at a critical time in their professional development.

### Emphasizing interprofessional education

Recent reports [13] have called for renewed emphasis on interprofessional education as an effective means of enhancing health professions education. However, to our knowledge, there has been limited research exploring how to implement interprofessional training in African settings. We assert that interprofessional training is the kind of transformative educational resource that can break down professional silos while also enhancing collaborative and non-hierarchical relationships that have undermined HIV care in many high burden settings. We recognize that implementing effective interprofessional education can only occur with the buy-in of institutional leadership and with sufficient funding, and we were fortunate to have financial support from HRSA and enthusiastic endorsement of deans of nursing and medical schools across all participating countries. Nonetheless, with institutional support and strong local leadership, this approach to learning can be adapted and implemented at minimal cost and is well suited to resource-constrained settings.

Implementing effective IPE also demands availability of faculty competent to lead interprofessional trainings, appropriate ratios of teachers to students, and coordination across professional training programs and academic calendars. Nonetheless, the necessity of this kind of instructional approach cannot be overstated. Not only is IPE important to ensuring high quality team-based HIV care, it is essential to the clinical practice of most major diseases in increasingly complex health settings in SSA. Given the scarcity of pedagogical resources for HP in Africa, IPE training modalities offer an efficient way to teach key domains of clinical practice to learners from across different cadres. Moreover, this kind of approach to education for health professions, matching competencies that correspond to local needs, offers a template for how elements of clinical training can be delivered sustainably. Our analysis highlights how this is feasible for training institutions to implement and also acceptable to educators to teach.

### Tools for quality improvement

Over the last two decades, there has been increasing recognition of the importance of quality improvement (QI) modalities to enhance HIV service delivery in SSA [13]. Simultaneously, quality management systems are being hardwired into health systems across the continent in order to optimize services, especially where services are constrained. Unfortunately, as one recent report highlighted, low quality care in Africa has a profound, deleterious impact and is responsible for millions of deaths each year [14]. In addition, our own needs assessment illustrated how few pre-service institutions taught QI modalities to health professions students. Although this curricular intervention provides learners with an introduction to a variety of QI tools, there remains a pressing need to weave this type of QI training into a more comprehensive approach in health professions training in SSA. Furthermore, greater HPE research in SSA is warranted to demonstrate how equipping learners with QI skills can be an effective catalyst to address inequities in access and quality of care. A key limitation of the intervention relates to the fact that it will be challenging to link its impact on clinical outcomes; feedback on the training and knowledge gained and retained are at best surrogate markers for clinical outcome measures. In the next phase of the project, an explicit goal is to determine the extent to which QI training leads to use of QI modalities in clinical practice that can lead to improvements in clinical outcomes. Last, in the next phase of the project, we intend to rigorously assess the impact of training on both learners and educators, collect qualitative and quantitative feedback from partner institutions to identify gaps in the original curriculum, and use evidence-based strategies to address them.

## Conclusion

Redesigning health professional education in SSA is necessary and timely, especially to ensure that previous investments made towards ending the HIV epidemic are not compromised. The STRIPE program is a model for collaborative, evidence-informed instructional design that was both feasible and acceptable to health professions educators in SSA. While further research is warranted to assess impact on knowledge and clinical outcomes, educational innovations such as the one described in this report, bringing an interprofessional transition course to SSA around a medical problem relevant to the region, offers a template for how curriculum development can be reimagined in way that is specific to Africa’s needs and resources.

## Supplementary Information

**Additional file 1.**

## Data Availability

All of the pedagogical content and training materials described in the manuscript are available at www.stripe.afrehealth.org

## Abbreviations

HRSA: Health and Resources Services Administration
HIV: Human immunodeficiency virus
IPE: Interprofessional education
IPC: Interprofessional collaboration
MEPI: Medical Education Partnership Initiative
NEPI: Nursing Education Partnership Initiative
PEPFAR: President’s Emergency Fund for AIDS Relief
QI: Quality improvement
SSA: Sub Saharan Africa
UCSF: University of California, San Francisco
