# Supplementary material for: Developing an interprofessional transition course to improve team-based HIV care for sub-Saharan Africa
Source: BMC Med Educ. 2020 Dec 9;20:499. doi: 10.1186/s12909-020-02420-x (PMC7725217; doi:10.1186/s12909-020-02420-x)
Supplement: Supplementary file 1 — Additional file 1. [file 12909_2020_2420_MOESM1_ESM.pdf]

Strengthening
Interprofessional
Education
for HIV

English

Open

This assessment is to be completed by all facilitators participating in STRIPE HIV. A participating facilitator is someone who received training by a partner institution and facilitated STRIPE HIV content to at least 1 group of learners. Please only proceed if this describes your engagement with STRIPE HIV.

(Select language in upper right/selecione o idioma no canto superior direito.)

Consent

Thank you for participating in STRIPE HIV. This program would not be possible without the investment from facilitators and for this we are grateful.

To better understand the impact of the STRIPE HIV workshops we are asking you to take part in a research study being done by a team at the University of California, San Francisco. If willing, kindly complete this electronic survey. The goal of this survey is to assess the impact of the STRIPE training on facilitators as well as learners. In addition, we welcome your suggestions for improvements and changes that can be made to enhance the impact of STRIPE HIV. The survey is intended to take less than 15-20 minutes, although you can stop it

at any time. If you consent, we may reach out and ask you to participate in another survey, focus group, or interview.

Participation in this study is optional. To protect the anonymity of respondents, we will not share your personal information, and only de-identified, aggregated responses will be shared outside the research team.

If you have any questions, please contact the STRIPE study team at STRIPEHIV@ucsf.edu. If you have questions or concerns about your rights as a research participant, you can contact the UCSF Institutional Review Board at +1 415-476-1814.

If you agree to participate in the survey, please check the accept box—please note that you will only have access to the questions if you accept.

- ○ Accept
- ○ Do not accept

Demographics

First Name

[ ]

Last Name

[ ]

Affiliation

Country

University Partner

Partner or Clinical Site

Health Profession Category

- ○ Medical
- ○ Nursing/Midwifery
- ○ Pharmacy
- ○ Laboratory
- ○ Other; specify:

Consider how you spend your time and assign percentages to the following roles (percentages should add to 100%):

Educating health professionals:	0
Clinical services:	0
Research (clinical, basic science, Implementation science, etc.):	0
Administration or other managerial responsibilities:	0
Other; specify:	0
Total	0

How many years ago did you graduate from health professional school?

- <5 years
- 5-9 years
- 10-15 years
- >15 years

Gender

- Female
- Male
- Other

Involvement

Please describe your engagement with STRIPE HIV:

Did you participate in a STRIPE HIV TOT (training of trainers)?

- Yes
- No

Were your provided the STRIPE HIV "Facilitator Guidelines for Leading Interprofessional Trainings?"

- Yes
- No
- I'm not sure

How many workshops did you participate in as a facilitator?

- ○ 1
- ○ 2
- ○ 3
- ○ >3

How many modules did you facilitate?

- ○ 1
- ○ 2
- ○ 3
- ○ 4
- ○ 5
- ○ >5

Please identify the STRIPE HIV modules that you facilitated or co-facilitated (select all that apply):

- ☐ 1: New HIV Diagnosis and ART Initiation in a Woman of Childbearing Age
- ☐ 2: Co-Morbidities in a Patient with Well Controlled HIV
- ☐ 3: Management of HIV-TB Co-Infection
- ☐ 4: PMTCT & Care for the Pregnant Woman with HIV
- ☐ 5: Care of the Adolescent Girl at Risk for HIV
- ☐ 6: Post-Exposure Prophylaxis
- ☐ 7: Care for the Patient with HIV & Cryptococcal Meningitis

- ☐ 8: Management of Sepsis in a Person with HIV
- ☐ 9: ART Adherence & Evaluation of Virologic Failure
- ☐ 10: End of Life Care in a Patient with HIV
- ☐ 11: Pre-Exposure Prophylaxis and Care for Men Who Have Sex with Men
- ☐ 12: Care for the Adolescent Male with Perinatally- Acquired HIV
- ☐ 13: Health System Building Blocks: Delivering High Quality Care to Patients with HIV
- ☐ 14: Community-Based HIV Service Delivery
- ☐ 15: Traditional and Complementary Medicine and Pneumocystis Pneumonia
- ☐ 16: Health Workforce Challenges and HIV Care Delivery
- ☐ 17: Care for the Paediatric Patient with HIV

Select all responses that describe your interest in continued engagement with STRIPE HIV:

- ☐ Incorporating STRIPE HIV Interprofessional Education (IPE) content into my **clinical practice**
- ☐ Incorporating STRIPE HIV IPE content into my **teaching**
- ☐ Incorporating Quality Improvement (QI) content into my **clinical practice**
- ☐ Incorporating QI content into my **teaching**
- ☐ Receiving additional training on IPE
- ☐ Receiving additional training in QI
- ☐ Receiving additional training in HIV
- ☐ Developing an international community of practice

Prior Engagement

Prior to your engagement with STRIPE HIV, to what extent:

	Not at all	A small amount	A moderate amount	A great deal
Had you **taught/trained** students from different health professions together in an interprofessional (IP) format?	○	○	○	○
Had you **received training** on **how to teach** different health professionals in an IP format?	○	○	○	○
Had you **received training** in training/courses that were **taught in** an IP format?	○	○	○	○
Were QI methodologies covered in the courses/training **you taught**?	○	○	○	○
Were QI methodologies covered in the courses/training that **you received**?	○	○	○	○
Had you **practiced** IP, team-based, patient-centered care in your engagement with HIV service delivery?	○	○	○	○
Had you **implemented** QI methodologies in the care you provide?	○	○	○	○

After your engagement with STRIPE HIV, to what extent:

	Not at all	A small amount	A moderate amount	A great deal
Do you plan to integrate HIV-related **IPE** in the courses/training **you teach?**	○	○	○	○
Do you plan to integrate **QI** methodologies in the HIV courses/training **you teach**?	○	○	○	○
Do you plan to integrate **IP collaborative practice** into the HIV clinical care **you provide**?	○	○	○	○

	Not at all	A small amount	A moderate amount	A great deal
Do you plan to integrate **QI** methodologies into the HIV clinical care **you provide**?	○	○	○	○

Optional: Related to your answers above, list any barriers you anticipate in integrating IPE and/or QI into the courses **you teach**.

Optional: Related to your answers above, list any barriers you anticipate in integrating IPE and/or QI into **your clinical practice**.

Knowledge and Skills, Comfort and Confidence

The modules you facilitated are shown below. Please assess your knowledge and skills related to each module's topic BEFORE and AFTER your involvement with STRIPE HIV.

BEFORE		AFTER

BEFORE					AFTER			
**No** knowledge or skills	**Limited** knowledge or skills	A **moderate amount** of knowledge or skills	**A lot** of knowledge or skills		**No** knowledge or skills	**Limited** knowledge or skills	A **moderate amount** of knowledge or skills	A kn[illegible] o[illegible]
O	O	O	O	Module 1: Assessing and managing a woman newly diagnosed with HIV using a team-based approach	O	O	O	
O	O	O	O	Module 2: Evaluating, preventing, and managing cardiometabolic complications of people with HIV and chronic disease management using a team-based approach	O	O	O	
O	O	O	O	Module 3: Providing team-based care for a patient with HIV and pulmonary TB	O	O	O	

BEFORE					AFTER			
**No** knowledge or skills	**Limited** knowledge or skills	A **moderate amount** of knowledge or skills	**A lot** of knowledge or skills		**No** knowledge or skills	**Limited** knowledge or skills	A **moderate amount** of knowledge or skills	**A** kn[illegible] o[illegible]
O	O	O	O	Module 4: Managing the care of pregnant women, new mothers and newborns living with HIV using an integrated approach to service delivery	O	O	O	
O	O	O	O	Module 5: Meeting the unique aspects of adolescent girls at risk for HIV using a team-based approach	O	O	O	
O	O	O	O	Module 6: Assessing and managing any colleague who presents with a potential work-related exposure to HIV	O	O	O	

BEFORE					AFTER			
**No** knowledge or skills	**Limited** knowledge or skills	A **moderate amount** of knowledge or skills	**A lot** of knowledge or skills		**No** knowledge or skills	**Limited** knowledge or skills	A **moderate amount** of knowledge or skills	[illegible]
O	O	O	O	Module 7: Providing team-based care for a patient with cryptococcal meningitis	O	O	O	
O	O	O	O	Module 8: Providing team-based care for a patient newly diagnosed with HIV who is admitted to the hospital with bacterial sepsis	O	O	O	
O	O	O	O	Module 9: Using evidence-based strategies to provide team-based care for a patient with ART non-adherence	O	O	O	

BEFORE					AFTER			
**No** knowledge or skills	**Limited** knowledge or skills	A **moderate amount** of knowledge or skills	**A lot** of knowledge or skills		**No** knowledge or skills	**Limited** knowledge or skills	A **moderate amount** of knowledge or skills	**A** kn o
○	○	○	○	Module 10: Providing compassionate, person-centered end of life care for patients with HIV with severe life-threatening diseases	○	○	○	
○	○	○	○	Module 11: Providing and promoting equitable, non-judgmental HIV services to key populations	○	○	○	
○	○	○	○	Module 12: Providing care to an adolescent male with perinatally-acquired HIV using a team-based approach	○	○	○	

BEFORE					AFTER			
**No** knowledge or skills	**Limited** knowledge or skills	A **moderate amount** of knowledge or skills	**A lot** of knowledge or skills		**No** knowledge or skills	**Limited** knowledge or skills	A **moderate amount** of knowledge or skills	[illegible]
○	○	○	○	Module 13: Understanding the building blocks of the health system and how they impact the care of patients with HIV	○	○	○	
○	○	○	○	Module 14: Understanding community-based, differentiated care delivery models available to patients with HIV	○	○	○	
○	○	○	○	Module 15: Providing person-centered care for a patient with an opportunistic pneumonia who is interested in traditional and complementary medicine	○	○	○	

BEFORE					AFTER			
**No** knowledge or skills	**Limited** knowledge or skills	A **moderate amount** of knowledge or skills	**A lot** of knowledge or skills		**No** knowledge or skills	**Limited** knowledge or skills	A **moderate amount** of knowledge or skills	**A** kn o
O	O	O	O	Module 16: Recognizing human resource challenges faced in delivering high quality care	O	O	O	
O	O	O	O	Module 17: Caring for a pediatric patient with HIV using a team-based approach	O	O	O	

The topics for the modules you facilitated are shown below. Consider whether STRIPE HIV improved your **confidence** in your **ability to teach** on these topics.

	No improvement	Minimal improvement	Moderate improvement	Significant improvement
Assessing and managing a woman newly diagnosed with HIV using a team-based approach	O	O	O	O

	No improvement	Minimal improvement	Moderate improvement	Significant improvement
Evaluating, preventing, and managing cardiometabolic complications of people with HIV and chronic disease management using a team-based approach	○	○	○	○
Providing team-based care for a patient with HIV and pulmonary TB	○	○	○	○
Managing the care of pregnant women, new mothers and newborns living with HIV using an integrated approach to service delivery	○	○	○	○
Meeting the unique aspects of adolescent girls at risk for HIV using a team-based approach	○	○	○	○
Assessing and managing any colleague who presents with a potential work-related exposure to HIV	○	○	○	○
Providing team-based care for a patient with cryptococcal meningitis	○	○	○	○
Providing team-based care for a patient newly diagnosed with HIV who is admitted to the hospital with bacterial sepsis	○	○	○	○
Using evidence-based strategies to provide team-based care for a patient with ART non-adherence	○	○	○	○

	No improvement	Minimal improvement	Moderate improvement	Significant improvement
Providing compassionate, person-centered end of life care for patients with HIV with severe life-threatening diseases	○	○	○	○
Providing and promoting equitable, non-judgmental HIV services to key populations	○	○	○	○
Providing care to an adolescent male with perinatally-acquired HIV using a team-based approach	○	○	○	○
Understanding the building blocks of the health system and how they impact the care of patients with HIV	○	○	○	○
Understanding community-based, differentiated care delivery models available to patients with HIV	○	○	○	○
Providing person-centered care for a patient with an opportunistic pneumonia who is interested in traditional and complementary medicine	○	○	○	○
Recognizing human resource challenges faced in delivering high quality care	○	○	○	○
Caring for a pediatric patient with HIV using a team-based approach	○	○	○	○

Principles of IP collaborative practice are listed below. Depending on the modules you facilitated, you may or may not have encountered these domains. Consider whether STRIPE

HIV improved your **confidence** in your **ability to teach** each of the following principles.

	No improvement	Minimal improvement	Moderate improvement	Significant improvement
Patient involvement in decision-making related to their care plans	○	○	○	○
Development of trusting relationships with patients and families	○	○	○	○
Roles and responsibilities of different health professionals	○	○	○	○
Working together as an interprofessional team to provide care	○	○	○	○
Communications tools to facilitate discussions that enhance team functioning	○	○	○	○
Influence of authority and hierarchy on team functioning	○	○	○	○
Leadership practices that support collaborative practice and team effectiveness	○	○	○	○
Integration of the knowledge and experiences of other professions to inform care decisions	○	○	○	○
Conflict resolution between interprofessional team members	○	○	○	○
Addressing complex medical ethical dilemmas as a member of an interprofessional team	○	○	○	○

Domains of QI are listed below. Depending on the modules you facilitated, you may or may not have encountered these domains. Consider whether STRIPE HIV improved your

confidence in your **ability to teach** each of the following domains.

	No improvement	Minimal improvement	Moderate improvement	Significant improvement
Identifying root causes of problems	○	○	○	○
Responding to patient-level problems to improve outcomes	○	○	○	○
Responding to systems-level problems to improve outcomes	○	○	○	○
Improving effectiveness of communication between members of the health care team	○	○	○	○
Using patient data to assess quality of care	○	○	○	○

Please rate how **confident** you are in your **ability to implement** the following areas:

	Not at all confident	Moderately confident	Extremely confident
Facilitating the workshop as part of a team of interprofessional educators	○	○	○
Training different types of health profession students together on HIV interprofessional collaboration	○	○	○
Integrating HIV IPE content into your health professions' education program outside of STRIPE HIV	○	○	○

Preparedness/relevance/material

Please select from the answer options below:

	Agree	Disagree
I felt prepared to facilitate the modules to an audience of interprofessional learners	○	○
I felt prepared to facilitate the QI content of the modules	○	○
I clearly understood the goals and objectives of the modules that I facilitated	○	○
I felt understood and respected by health educators of other professions	○	○
The content was relevant to learners	○	○
Participating in STRIPE advanced my own knowledge and skills	○	○
I had access to the tools and materials that I needed to successfully facilitate the workshop	○	○
The facilitator guides were informative and easy to use	○	○

Short Answers

Thank you for your time. Before ending the survey, please consider providing brief responses to the following questions:

Optional: What aspect of STRIPE HIV did you enjoy the most?

Optional: What aspect of STRIPE HIV did you enjoy the least?

Optional: What is the most important element you take away from this experience?

Optional: What impact do you think this training has on learners?

Optional: What impact do you think this training has on facilitators?

Optional: What suggestions do you have to improve STRIPE HIV and increase impact?

Optional: Do you think that STRIPE HIV should be more widely implemented at your institution?

- ○ Yes
- ○ No

Optional: Please explain why or why not.

This is the end of the survey. Enter any additional feedback or comments you have.

Closing

Do you give permission to be contacted for any follow up assessments, key informant interviews, or focus groups?

- ○ [ ] Yes; enter email address:
- ○ No

Powered by Qualtrics
